# Modulation of the Functions of Goat Peripheral Blood Mononuclear Cells by *Fasciola gigantica* Thioredoxin Peroxidase In Vitro

**DOI:** 10.3390/pathogens9090758

**Published:** 2020-09-17

**Authors:** Ai-Ling Tian, Xiaowei Tian, Dan Chen, Mingmin Lu, Guillermo Calderón-Mantilla, Xiao-Dan Yuan, Xiangrui Li, Hany M. Elsheikha, Xing-Quan Zhu

**Affiliations:** 1 State Key Laboratory of Veterinary Etiological Biology, Key Laboratory of Veterinary Parasitology of Gansu Province, Lanzhou Veterinary Research Institute, Chinese Academy of Agricultural Sciences, Lanzhou 730046, China; ailingtian@hotmail.com (A.-L.T.); chendan0623@hotmail.com (D.C.); yuanxiaodan0118@outlook.com (X.-D.Y.); 2 College of Veterinary Medicine, Nanjing Agricultural University, Nanjing 210095, China; tianxw2020@163.com (X.T.); lumingmin2008@hotmail.com (M.L.); lixiangrui@njau.edu.cn (X.L.); 3 Facultad de Ingeniería, Universidad de La Sabana, Campus del Puente del Común, Km. 7, Autopista Norte de Bogotá. Chía, Cundinamarca 140013, Colombia; guillermo.calderon@unisabana.edu.co; 4 Faculty of Medicine and Health Sciences, School of Veterinary Medicine and Science, University of Nottingham, Sutton Bonington Campus, Loughborough LE12 5RD, UK; 5 College of Veterinary Medicine, Shanxi Agricultural University, Jinzhong 030801, China

**Keywords:** *Fasciola gigantica*, thioredoxin peroxidase, immunoregulation, peripheral blood mononuclear cells, immune responses

## Abstract

The liver fluke *Fasciola gigantica* has a remarkable ability to establish a long-term infection within the hepatobiliary system of the mammalian definitive host. *F. gigantica* achieves this by producing excretory–secretory molecules, which have immunomodulatory activities. In an effort to elucidate the immunomodulatory functions of *F. gigantica* thioredoxin peroxidase protein (FgTPx), we expressed recombinant FgTPx (rFgTPx) in *Escherichia coli* bacteria and examined its effects on several functions of goat peripheral blood mononuclear cells (PBMCs) in vitro. Sequence analysis revealed that FgTPx is related to a thioredoxin-like superfamily. Western blot analysis showed that rFgTPx was recognized by the sera of goats experimentally infected by *F. gigantica*. The specific binding of rFgTPx protein to the surface of goat PBMCs was demonstrated by immunofluorescence staining. We investigated the influence of serial concentrations of rFgTPx on various functions of goat PBMCs. All concentrations of rFgTPx increased the secretion of interleukin-2 (IL-2), IL-4, IL-10, IL-17, transforming growth factor-beta (TGF-β), and interferon gamma (IFN-γ), but inhibited PBMC proliferation, migration, and monocyte phagocytosis. Goat PBMCs exposed to 20–40 μg/mL of rFgTPx secreted increased levels of nitric oxide (NO), and 10–40 μg/mL of rFgTPx promoted cell apoptosis. These findings indicate that rFgTPx influences various functions of goat PBMCs by interacting with a large number of cellular targets, ultimately to promote the parasite’s survival. The roles of rFgTPx and their interacting proteins warrant further investigation.

## 1. Introduction

Fasciolosis is a zoonotic parasitic disease caused by the liver flukes *Fasciola gigantica* and *Fasciola hepatica* [1]. The estimated economic loss every year due to fasciolosis is over 3 billion US dollars worldwide [2]. Also, fasciolosis has been listed by the World Health Organization (WHO) as one of the neglected tropical human diseases [3,4,5]: at least 240 million people worldwide are readily infected, and 180 million people are at risk of infection [6]. Given this serious impact on both the human health and sustainable agricultural economy, and because of the increase of anthelmintic resistance, alternative control strategies, such as vaccination and understanding the genetic basis of the host’s innate resistance, have received increasing attention [7,8,9]. Some immunoprotective antigens have been identified, such as the immunoglobulin G subclass and fatty acid binding protein [10,11,12,13]. Despite these efforts, no commercial vaccine is available as yet. More understanding of the molecular mechanism of *F. gigantica* infection can provide new insight into the protective immune responses, which ultimately can facilitate the development of new immunoregulatory therapeutic interventions.

Liver flukes of the genus *Fasciola* generally cause chronic (long-term) infections and survive well inside their host, which has been attributed to the parasite’s ability to evade immune defense mechanisms [14,15,16]. Excretory and secretory products (ESPs), containing many active molecules, have been produced by these parasites under in vitro and in vivo conditions [17]. Previous reports showed that different molecules of ESPs from *F. gigantica* (FgESPs) can cause various immune responses—for example, some molecules can simulate host defense against parasites, while other have exerted the opposite effect [16,18,19,20,21]. Of particular importance to their survival is the ability of liver flukes to employ an anti–oxidant, redox-based system that protects the liver flukes against the reactive oxygen and nitrogen species produced by host immune cells and endogenous cellular metabolism [22]. Thioredoxin (FhTRx) and the related protein peroxiredoxin (FhPRx), key components of this anti–oxidant system, were amongst the most abundant proteins detected in the secretome of newly excysted juveniles (NEJ) of *F. hepatica* [23]. 

Our preliminary proteomic studies have indicated the potential immunomodulatory roles of thioredoxin peroxidase (TPx), one of the ESPs of *F. gigantica*, by revealing its ability to bind to peripheral blood mononuclear cells (PBMCs) isolated from infected goats (unpublished data). However, the effect of *F. gigantica* TPx (FgTPx) on the functions of goat PBMCs is unknown. In the present study, we cloned and expressed the *FgTPx* gene, and explored the outcomes of exposing goat PBMCs to serial concentrations of recombinant FgTPx (rFgTPx) on various functions of goat PBMCs in vitro. Our data revealed diverse effects of rFgTPx on the functions of goat PBMCs, which seem to promote the parasite’s survival and establishment of a persistent infection. These results present rFgTPx as a new potential target for the development of immunomodulatory interventions for the control of *F. gigantica* infection.

## 2. Results

### 2.1. De Novo Prediction of FgTPx Protein

**Figure 1:**
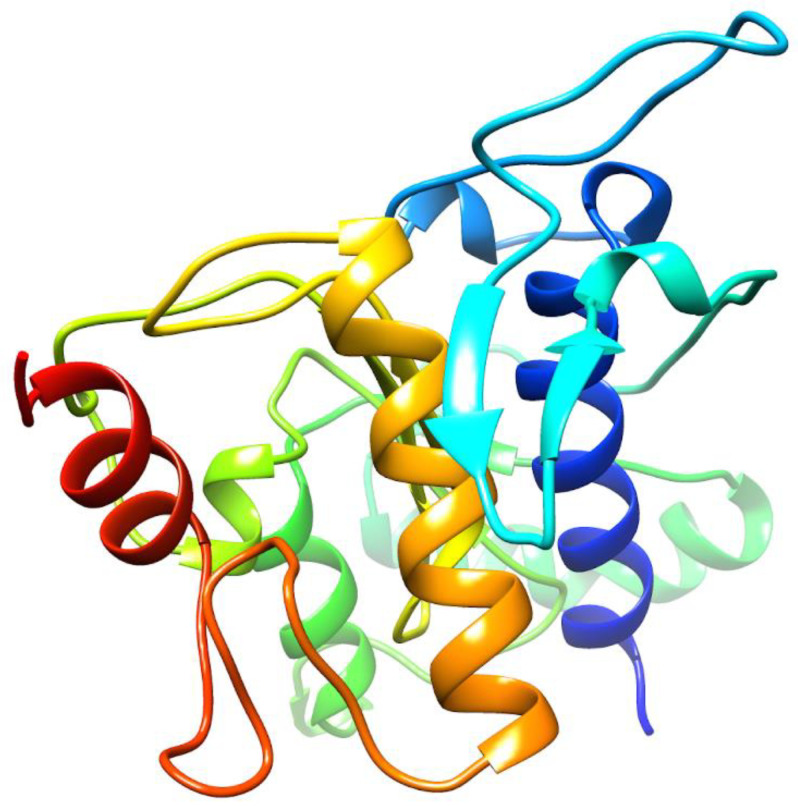
De novo three-dimensional (3D) model of *Fasciola gigantica* thioredoxin peroxidase (FgTPx) protein, constructed based on the ab initio method of protein modeling using Rosetta.

Protein blast results showed that the FgTPx protein is homologous to human peroxiredoxin–2 (PRDX2), with an identity of 62%, an E–value of 1e–75, a query cover of 78%, and a max score of 229. The human PRDX2 protein is a 197-amino-acid protein, with a molecular mass of ~21.9 KDa (Uniprot ID: P32119). Rosetta-based prediction was used to generate a de novo model of FgTPx protein (Figure 1).

### 2.2. Cloning and Sequence Analysis of FgTPx

The PCR product corresponding to the *FgTPx* gene (~711 bp) was obtained and successfully cloned into a pMD19-T cloning vector. Nucleic acid sequencing showed that the positive clones of pMD19–T/*FgTPx* contained an insert of 711 bp. The sequence of *FgTPx* gene encodes a protein of 236 amino acids, with a molecular mass of 26.57 kDa and a theoretical isoelectric point (pI) of 8.01. Conserved domains analysis showed that the protein belonged to a thioredoxin-like super family. Amino acid sequence alignment of *FgTPx* showed 100% homology with the corresponding region of *F. gigantica* sequence available in the National Center for Biotechnology Information (NCBI) database (GenBank accession no. ABY85785.1).

### 2.3. Expression, Purification, and Detection of rFgTPx

**Figure 2:**
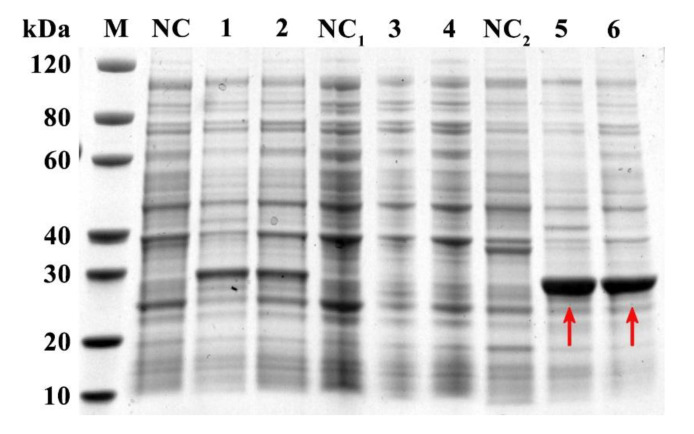
SDS–PAGE analysis of recombinant FgTPx (rFgTPx). Lane M: protein molecular weight marker in kDa; Lane NC: bacterial cell lysate without induction; Lane 1: cell lysate with induction for 16 h at 15 °C; Lane 2: cell lysate with induction for 4 h at 37 °C; Lane NC1: supernatant of cell lysate without induction; Lane 3: supernatant of cell lysate with induction for 16 h at 15 °C; Lane 4: supernatant of cell lysate with induction for 4 h at 37 °C; Lane NC2: pellet of cell lysate without induction; Lane 5: pellet of cell lysate with induction for 16 h at 15 °C; Lane 6: pellet of cell lysate with induction for 4 h at 37 °C. The red arrows denote the recombinant protein (rFgTPx).

**Figure 3:**
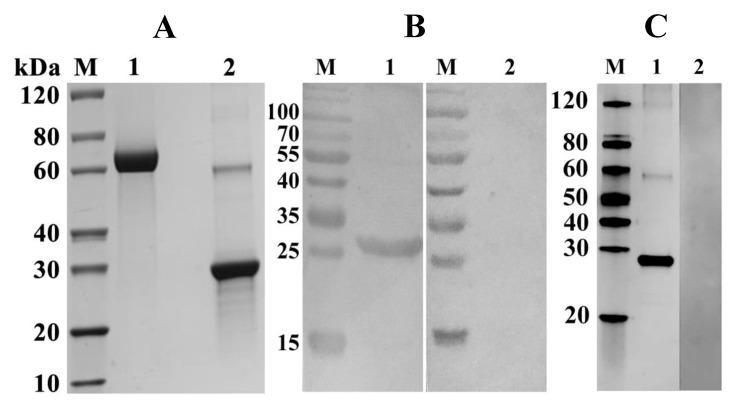
SDS–PAGE and Western-blot analyses of the purified recombinant protein, and rFgTPx from the sonicated pellet of *E. coli*. (**A**) Proteins were resolved on 12% acrylamide gels and stained with Coomassie brilliant blue R250. Lane M: protein molecular weight marker in kDa; Lane 1: 5 μg bovine serum albumin; Lane 2: purified rFgTPx appeared as a single band of ~26 kDa. (**B**,**C**) The protein of interest was run under non–reducing conditions and visualized using a chemiluminescent horseradish peroxidase substrate. Lane M: protein molecular weight marker in kDa. (**B**) Lane 1 was loaded with rFgTPx. Serum from *F. gigantica*–infected goats detected a single band of ~26 kDa; Lane 2 was loaded with rFgTPx that did not react with serum of uninfected goat. (**C**) Lane 1 was loaded with rFgTPx and incubated with rabbit serum containing specific anti-rFgTPx antibodies, showing a single band at ~26 kDa band; Lane 2 was loaded with rFgTPx that did not react with naïve rabbit serum.

The fragment of *FgTPx* gene was successfully cloned into pET–28a(+) vectors, and the positive clones were designated as pET–28a/*FgTPx*. Protein expression was induced by isopropyl–β–d–thiogalactopyranoside (IPTG) in an *E. coli* BL21 (DE3) strain. The recombinant protein (rFgTPx) was expressed as His-tagged fusion proteins that were mainly produced as inclusion bodies (Figure 2). After purification, the concentrated protein was detected with a molecular mass ~ 26 kDa on the SDS–PAGE gel (Figure 3A). Western blot analysis showed that rFgTPx migrated at about 26 kDa and could be recognized by anti–*F. gigantica* serum from experimentally infected goats, but did not react with the serum of an uninfected goat (Figure 3B). The rFgTPx was also detected using rabbit serum containing specific anti-rFgTPx antibodies, but was not reactive to naïve rabbit serum (Figure 3C).

### 2.4. Immunofluorescence Assay

**Figure 4:**
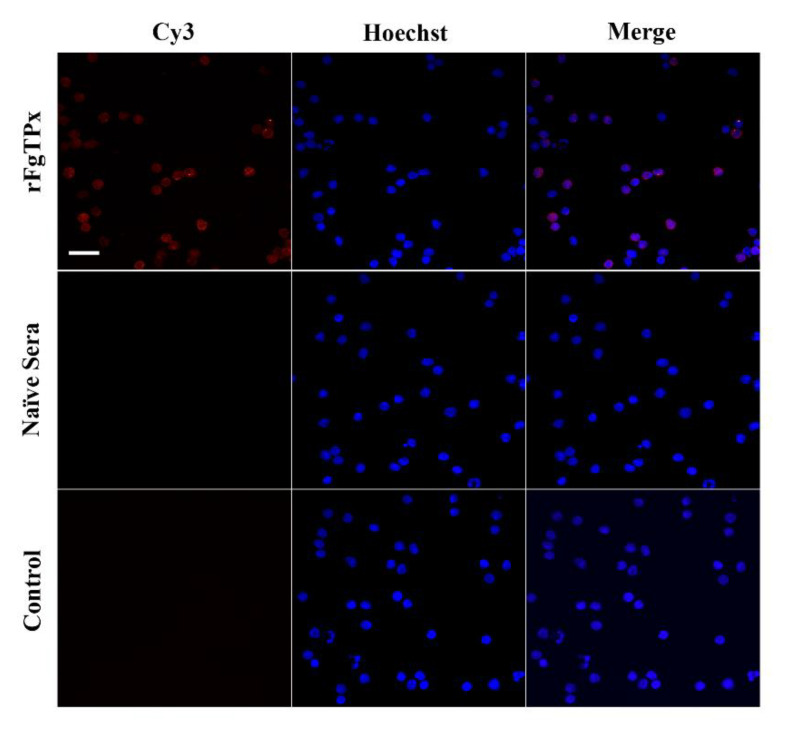
*Fasciola gigantica*–derived rFgTPx binds to the surface of goat PBMCs. Visualization of rFgTPx attachment to PBMC surfaces was carried out by incubation of PBMCs treated or untreated with rFgTPx with rabbit anti–rFgTPx primary antibody. Hoechst (blue) and Cy3-conjugated secondary antibody (red) were used to stain host cell nuclei and rFgTPx, respectively. Positive staining of cell surface was detected in rFgTPx–treated cells only. No staining was detectable in control, untreated cells. Scale bars = 10 µm.

Cultured goat PBMCs incubated with rFgTPx were examined by immunofluorescence assay (IFA) using confocal microscopy (40× magnification). As shown in Figure 4, in the presence of the recombinant protein, the rabbit-derived anti-rFgTPx antibodies stained the PBMCs. On the contrary, no fluorescence was detected in non-treated control cells. The dense concentration of red color around PBMCs suggests that rFgTPx binds to the cell surface.

### 2.5. Cytokine Production, Cell Proliferation, Migration, and Production of NO

**Figure 5:**
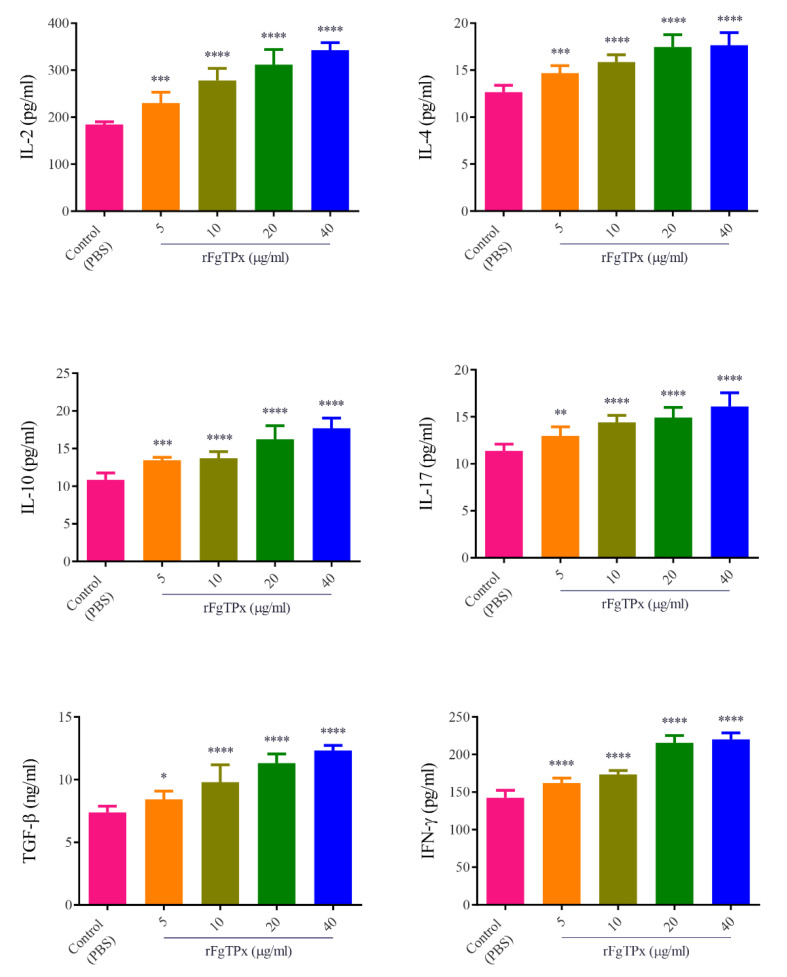
The rFgTPx induced polarized patterns of cytokine secretion. Goat PBMCs were incubated for 72 h in the presence or absence of the indicated concentrations of rFgTPx (µg/mL). The levels of cytokine concentration in the supernatant of cultured PBMCs was quantified by ELISA. Graphs represent means ± standard deviations of the results from three independent experiments. Asterisks indicate statistical significance between treated and untreated control goat PBMCs (* *p* < 0.05; ** *p* < 0.01; *** *p* < 0.001; **** *p* < 0.0001).

**Figure 6:**
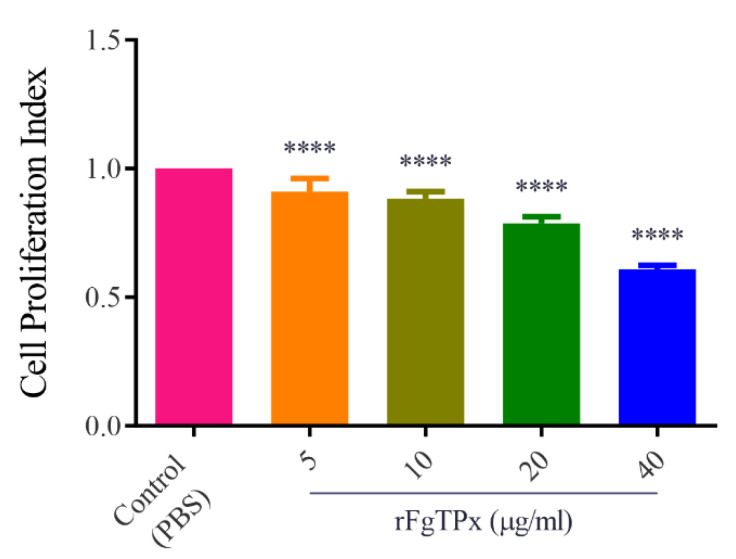
The rFgTPx inhibits goat PBMC proliferation. Goat PBMCs were sham-treated with phosphate-buffered saline (PBS) or with the indicated concentrations of rFgTPx (µg/mL) and incubated for 48 h at 37 °C. Proliferation of cells was determined using the cell counting Kit-8 (CCK-8) assay. The rFgTPx significantly inhibited PBMC proliferation. Graphs represent means ± standard deviations of results from three independent biological replicates. Asterisks indicate statistical significance between treated cells and control cells (**** *p* < 0.0001).

**Figure 7:**
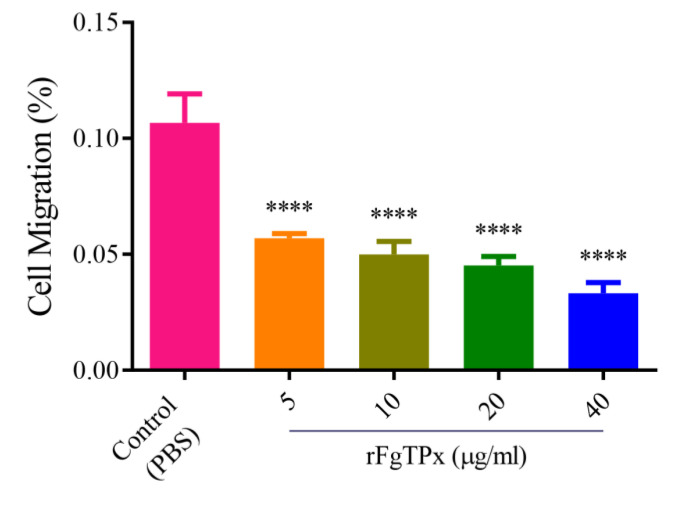
The rFgTPx suppressed goat PBMC migration. Goat PBMCs were sham-treated with PBS or with the shown concentrations of rFgTPx, then cell migration percentage (%) was determined. Graphs represent means ± standard deviations of data from three independent experiments. The asterisks indicate significant difference between treated and sham-treated control cells (**** *p* < 0.0001).

**Figure 8:**
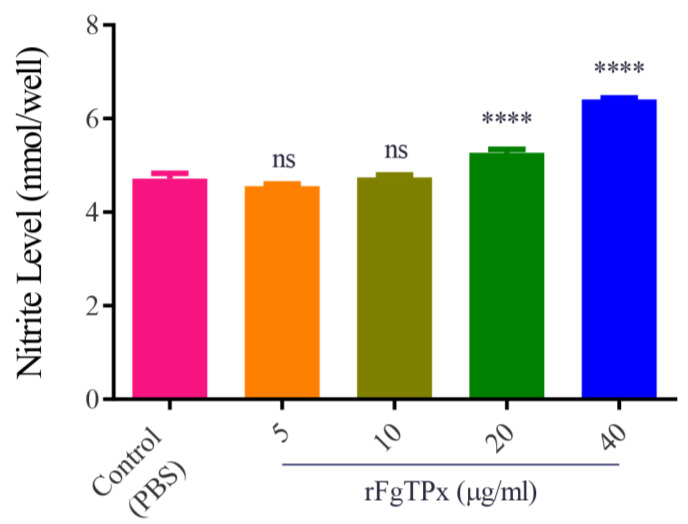
Effect of rFgTPx on the intracellular total NO production. Goat PBMCs were sham-treated with PBS or the indicated concentrations of rFgTPx and maintained at 37 °C. Total NO concentration produced by PBMCs was measured by Griess assay. Graphs represent means ± standard deviations of data from three independent experiments. Asterisks indicate significant difference between treated and non-treated control cells (**** *p* < 0.0001; ns: non-significant).

We investigated the potential modulatory effect of rFgTPx on PBMCs. As shown in Figure 5, when cells were treated with rFgTPx (5, 10, 20, and 40 μg/mL), the production of IL-2, IL-4, IL-10, IL-17, IFN-γ, and TGF-β were significantly increased compared to the control, untreated cells. Cell proliferation was significantly inhibited in cells treated with rFgTPx in a dose–dependent manner (Figure 6). All concentrations—5 μg/mL, 10 μg/mL, 20 μg/mL, and 40 μg/mL—of rFgTPx significantly suppressed the migration of treated cells in a dose-dependent manner, compared with the control cells (Figure 7). As showed in Figure 8, total NO release from the cells was significantly increased in the presence of rFgTPx at 20 μg/mL and 40 μg/mL, compared to the control, but not at 5 μg/mL and 10 μg/mL.

### 2.6. Monocyte Phagocytosis and Apoptosis

**Figure 9:**
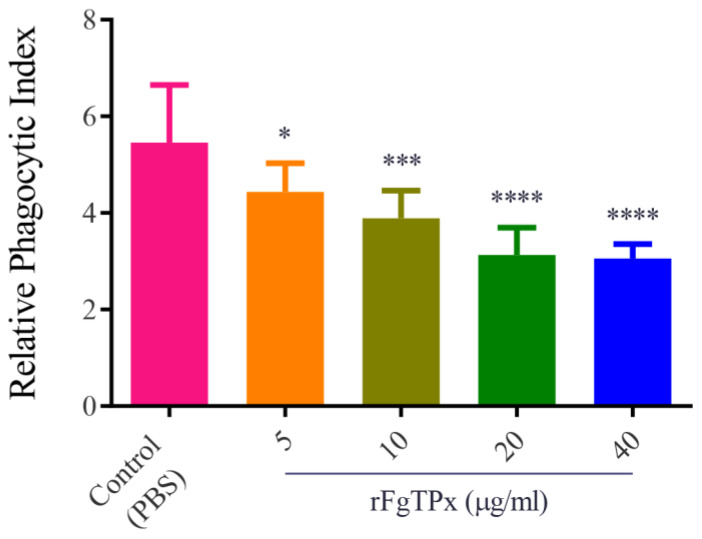
The rFgTPx inhibited the phagocytic ability of monocytes, as indicated by the reduction in FITC–dextran uptake in a dose-dependent manner. Graphs represent means ± standard deviations of data from three independent experiments. Significance was set at * *p* < 0.05; *** *p* < 0.001, and **** *p* < 0.0001 compared to sham-treated monocytes.

**Figure 10:**
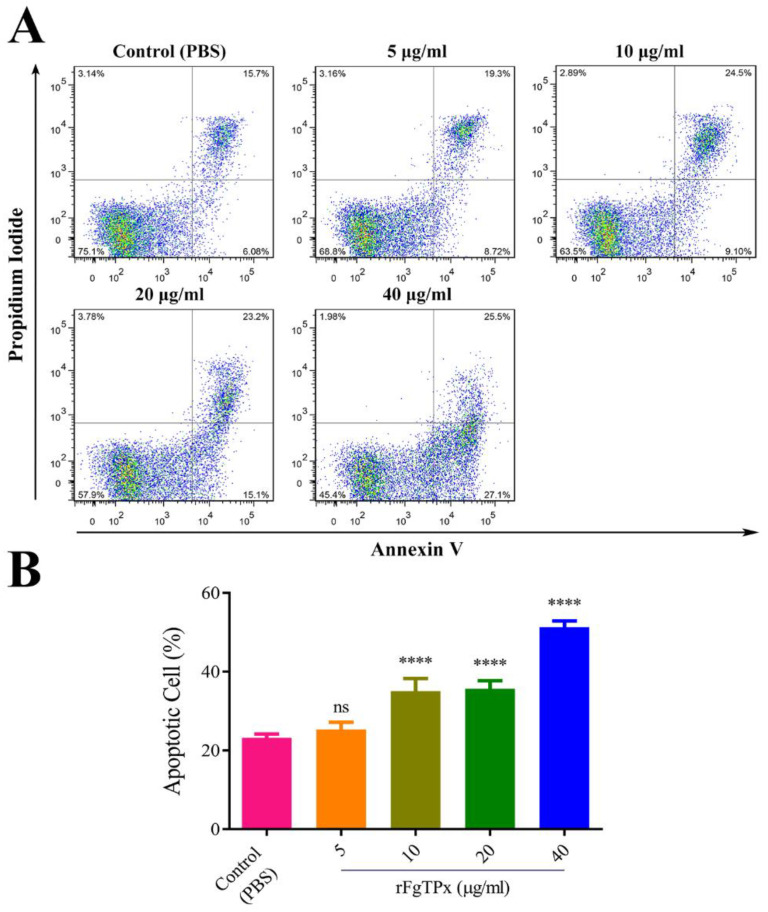
The rFgTPx induced apoptosis in goat PBMCs. Apoptotic cells were determined by annexin V/PI staining and flow cytometry. (**A**) Flow cytometric analysis shows the death of goat PBMCs in response to exposure to increasing concentrations of rFgTPx. (**B**) The percentage of apoptotic cells (annexin V+/PI–) compared to total cell population. Graphs represent means ± standard deviations of data from three independent experiments. The asterisks indicate statistically significant differences between treated and untreated control PBMCs (**** *p* < 0.0001; ns: non-significant).

The cell phagocytosis was examined by uptaking fluorescein isothiocyanate (FITC)–dextran. Results revealed that in rFgTPx induced a significant reduction in monocyte phagocytosis (Figure 9). The rFgTPx seems to have a proapoptotic effect. We detected a correlation between PBMC exposure to different concentrations of rFgTPx and the percentage of apoptotic cells. As shown in Figure 10, rFgTPx significantly induced apoptosis in goat PBMCs at 10 μg/mL, 20 μg/mL, and 40 μg/mL, compared to the control group, but not at 5 μg/mL.

## 3. Discussion

Given our interest in understanding the immunomodulatory activities of the ESPs of the liver fluke *F. gigantica*, we characterized one of the *F. gigantica* ESPs well-known for its antioxidant properties. The thioredoxin peroxidase (*TPx*) gene of *F. gigantica* was cloned and expressed in a prokaryotic expression system. Sequence analysis and Rosetta-based prediction revealed that FgTPx is related to the thioredoxin-like superfamily, which plays a role in the antioxidant defense system of the parasite. The expressed rFgTPx protein has a molecular mass of ~26 kDa, which was confirmed by Western blot analysis using sera from *F. gigantica*-infected goats. Using rabbit-derived, anti-rFgTPx antibody, we demonstrated the specific binding of rFgTPx to the surface of goat PBMCs. Further validation of the anti-rFgTPx antibody on *F. gigantica* extracts or fractions of *F. gigantica*-secreted proteins should be conducted in future studies, in order to gain more information on the physiological relevance of FgTPX binding to host cells.

It is readily known that other *F. gigantica* effector proteins, rFg14-3-3e [20] and rFgRab10 [21], alter the phagocytic function of monocytes by decreasing or increasing their phagocytic capacities, respectively, or simply kill PBMCs by inducing apoptosis. By reducing monocyte phagocytosis, rFgTPx would augment the action of rFg14-3-3e [20] and antagonize the action of rFgRab10 [21] with regard to the phagocytic capacity of monocytes. By promoting apoptosis at 10–40 μg/mL concentrations, rFgTPx seems to potentiate the action of both rFg14-3-3e and rFgRab10 proteins. The three proteins (rFgTPx, rFg14-3-3e and rFgRab10) exhibit a similar action, not only in regard to their proapoptotic activity, but also in the context of increasing NO production and reducing cell proliferation. Also, while rFgTPx and rFg14-3-3e suppress cell migration, rFgRab10 seems to exert the opposite effect on cell migration.

Liver flukes have developed a variety of mechanisms to counterbalance the antiparasitic potential of the innate immune effectors of their mammalian hosts. For example, thioredoxin (TRx) protein is one of the ESPs of *F. hepatica* (FhPRx) that serves as a pathogen-associated molecular pattern (PAMP), increases the induction and recruitment of alternatively activated (M2) macrophages, and skews the host immunity towards a Th2 type response [24]. In our study, rFgTPx attached with high affinity to the surface of PBMCs, indicating that this protein may interact with immune cell surface receptors, thereby inducing immune response. *F. gigantica* is a helminth parasite that drives Th1/Th2 immune responses in its mammalian host [25,26,27]. *F. gigantica* discharges many ES molecules that seem to play roles in the induction of this type of immune response. *F. gigantica*-derived ESPs, identified in the present study (rFgTPx) and in previous studies (rFg14-3-3e [20] and rFgRab10 [21]), have induced Th1 (IFN-γ, IL-2) and Th2 (IL-4, IL-10, TGF-β) cytokines, except IL-4 and IFN-γ, whose levels were low in response to rFg14-3-3e. Cytokines are produced by a variety of immune cells and play various roles in protecting the host and maintaining tissue homeostasis [28,29]. In an earlier study on *F. gigantica* infection in buffaloes, we observed that a mixed Th1/Th2 immune response is dominant from 28 to 70 dpi, to promote *F. gigantica* survival while limiting host tissue damage [27].

Th1 cells mainly secrete IL-2 and IFN-γ to control intracellular pathogens, and IFN-γ can activate monocytes [30], thereby promoting the release of NO [31,32]. The production of NO by a host’s immune system can damage the parasite [33,34]. Th2 cells mainly secrete IL-4, which mediates humoral immunity [35] and promotes antibody-dependent killing of eosinophils, macrophages, and mast cells [36,37,38]. Similar to rFg14-3-3e and rFgRab10, rFgTPx promoted apoptosis in goat PBMCs. Apoptosis has been reported in sheep peritoneal leucocytes during early *F. hepatica* infection to improve the survival of juvenile flukes during peritoneal migration to the liver [39]. Previous research has shown that apoptotic cells may be involved in inhibiting inflammatory responses through the induction of anti–inflammatory cytokines, such as IL-10 [40,41] and TGF-β [42,43]. Hence, both IL-10 and TGF-β cytokines are released from apoptotic goat PBMCs to counterbalance the proinflammatory response mediated by Th1 cytokines. Thioredoxin peroxidase from *F. hepatica* has also been shown to have antioxidant and immunosuppressive activity [44]. Our data support the hypothesis that rFgTPx modulates the immune response of PBMCs by promoting apoptosis, but further studies are needed to identify the involved signaling pathways and the precise molecular mechanism of PBMC apoptosis caused by rFgTPx protein. This phenomenon has also been observed in eosinophils exposed to ESPs from *F. hepatica*, suggesting that inducing apoptosis is a general mechanism used by liver flukes to manipulate and subvert the host immune system [45].

The increased level of the proinflammatory cytokine IL-17 indicates that IL-17 and Th17 contribute to immunoregulatory mechanisms during *F. gigantica* infection. In the course of our previous research, a modest Th2 response at the early stage of infection was found to balance harmful Th1 and Th17 responses in *F. gigantica*-infected buffaloes [46]. We also observed that *F. gigantica* infection had a significant impact on the expression of Th1, Th2, Th17, and Treg cytokines [47]. IL-17 is important in the host defense against extracellular pathogens, predominantly at mucosal sites. This cytokine can be generated by adaptive or innate immune cells and participates in host immune response against pathogens by increasing neutrophil expansion through modulating the expression of granulocyte colony-stimulating factor and by recruitment of immune cells to sites of inflammation via modulating the expression of the chemokine CXC [48,49].

Our findings suggest that while rFgTPx elicits a strong immune response and promotes the apoptosis of host cells, this protein also inhibits monocyte phagocytic function, and inhibits the proliferation and migration of PBMCs. These results suggest that rFgTPx may interfere with the differentiation and developmental pathway of PBMCs and suppresses their migration. These effects would result in a less effective immune response against *F. gigantica* by reducing the number of immune cells recruited to the infection sites, which could promote the parasite survival. Taken together, the diverse modulatory effects of rFgTPx on goat PBMCs suggest that rFgTPx protein interacts with various proteins/regulatory partners and modulates cellular processes to create niches that support the liver flukes’ persistence inside the host. However, caution must be taken in the interpretation of the results obtained in the present study, because the timing of secretion and the secreted amounts of FgTPx protein in vivo are unknown. Hence, these in vitro observations may not be completely functionally relevant in the context of a real infection.

## 4. Materials and Methods 

### 4.1. Ethics Approval

Experimental procedures performed in this study were reviewed and approved by the Animal Administration and Ethics Committee of Lanzhou Veterinary Research Institute, Chinese Academy of Agricultural Sciences. All animals were handled strictly according to the Animal Ethics Procedures and Guidelines of the People’s Republic of China.

### 4.2. Parasites and Animals

Adult *F. gigantica* flukes were obtained from Guangxi Zhuang Autonomous Region (China) as described previously [20]. Eight local goats (3 to 6 months old) were used in this study. As previously described, goats were treated with triclabendazole to rule out *Fasciola* infection and kept under hygienic *F. gigantica*-free conditions [21]. All goats were fed with commercial hay and corn, and allowed to drink water ad libitum. Female New Zealand Rabbits (4 months old) purchased from GenScript Biotech Corp (Nanjing, China) were used to produce antibodies. Rabbits were kept in specific pathogen-free (SPR) conditions and allowed access to sterilized food and water *ad libitum*.

### 4.3. Bioinformatis Analysis

To predict the de novo structure of the FgTPx sequence, we identified the most homologous protein (peroxiredoxin-2 protein (PRDX2), Uniprot ID: P32119) in humans using protein BLAST [50]. Then, we performed Rosetta-based prediction to generate a de novo 3D model [51].

### 4.4. Construction of Prokaryotic Expression Vector of FgTPx

Total RNA extraction from adult *F. gigantica* was performed using a single-step protocol, as previously described [29]. The complementary DNA (cDNA) was obtained with reverse transcription polymerase chain reaction (RT-PCR), using a cDNA Kit (TaKaRa Biotechnology, Dalian, Liaoning, China), according to the manufacturer’s instructions. The sequence of *FgTPx* gene was amplified using PCR and specific primers targeting the conserved domain sequences (CDSs) of *FgTPx* gene (GenBank accession no: ABY85785.1). Gene-specific primers (forward primer: 5′- CATATGCTCTCAAGTTCACTTATAATCG-3′, and reverse primer: 5′-CTCGAGGTTGGCTGAGGAGAAATA-3′) included *NdeI* and *XhoI* restriction sites (underlined). The *FgTPx* fragment was cloned into a pMD19–T vector (TaKaRa Biotechnology, Dalian, Liaoning, China), which was transformed into *Escherichia coli* DH5α cells using the CaCl_2_ transformation method. The positive clones were verified by double digestion and sequencing. The *FgTPx* gene was then sub-cloned into a pET–28a(+) vector (Invitrogen, Carlsbad, CA, USA), followed by transformation into *E. coli* BL21 (DE3) cells using the same transformation method. The constructed recombinant plasmid was thereafter named as pET–28a/*FgTPx*.

### 4.5. Expression and Purification of Recombinant FgTPx (rFgTPx)

The vector pET–28a encoding *FgTPx* were transformed to *E. coli* BL21 (DE3)-competent cells, and protein expression was induced with1 mM isopropyl–β–d–thiogalactopyranoside (IPTG; Sigma-Aldrich, USA). The purification of rFgTPx was performed as previously described. Briefly, the bacterial cells were harvested by centrifugation after 5 h post-IPTG induction. Following centrifugation at 10,000× *g*, the cells were sonicated and again centrifuged, and a Ni^2+^–nitrilotriacetic acid (Ni–NTA) column (GE Healthcare, Chicago, IL, USA) was used to purify the rFgTPx with His-tag, according to the manufacturer’s instructions. Buffer containing a linear, decreased urea gradient was used to dialyze the eluted protein, in order to be refolded and renatured. Then phosphate-buffered saline (PBS; pH 7.4) was used to exchange the urea-containing buffer. The endotoxin was removed from the protein samples using Detoxi-Gel Affinity Pak prepacked columns (Pierce Biotechnology Inc., Rockford, IL, USA). Then, the level of endotoxin of the recombinant protein (<1 EU per 1 mg) was measured by limulus amoebocyte lysate (LAL) gel clot assay using a Pyrosate Kit (Associates of Cape Cod Inc., East Falmouth, MA, USA). The purified recombinant protein was detected by 12% SDS-PAGE, and visualized by Coomassie blue staining, and its concentration was measured by using Bradford method.

### 4.6. Generation of Antibodies

The goat anti-sera used in immunoblot analysis were obtained from *F. gigantica*-infected goats. Four healthy goats (4–7 months old) were infected orally with 250 viable encysted metacercariae. After three months, the goat blood was collected, and serum containing anti-*F. gigantica* antibodies was separated. Naïve goat sera (used as negative control) were collected from a healthy goat and stored at –80 ℃ for later use. Specific antibodies against rFgTPx were prepared following the protocols described previously [20], except that rabbits were used in the present study. 

### 4.7. Western Blot Analysis

Western blot analysis was performed as previously described [21]. Briefly, the purified rFgTPx was loaded onto 12% SDS-PAGE gel, then transferred to Hybond-C extra nitrocellulose membranes (Amersham Biosciences, United Kingdom) and blocked with 5% bovine serum albumin (BSA) in TBS–Tween 20 (TBST) for 1 h at 37 °C. Goat antisera (1:100 dilution with TBST containing 5% BSA) or rabbit serum (1:200 dilution) were used as primary antibodies. Horseradish Peroxidase (HRP)-conjugated rabbit anti-goat immunoglobulin G (IgG) (Sigma, USA) (1:5000 dilution with TBST containing 5% BSA) or goat anti-rabbit IgG-HRP antibody (Invitrogen, USA) (1:10,000 dilution) were used as secondary antibodies. Western blot assay was developed with 3,3′-diaminobenzidine (DAB; Sigma), as a chromogenic substrate, to detect the immune reaction.

### 4.8. Isolation and Culture of Goat PBMCs and Monocytes

Goat PBMCs were isolated from the jugular vein of three healthy goats using the standard Ficoll–hypaque (GE Healthcare, USA) and gradient centrifugation, as previously described [52]. The monocytes were isolated from the PBMCs based on their adhesion to plastic plates. Finally, PBMCs and monocytes were respectively suspended to a final density of 1 × 10^6^ cells/mL in Roswell Park Memorial Institute (RPMI) 1640 or Dulbecco’s Modified Eagle Medium (DMEM) medium, containing 10% heat-inactivated fetal bovine serum (FBS), 100 U/mL penicillin, and 10,000 U/mL penicillin and streptomycin (Gibco, Grand Island, NY, USA). Cell viability were examined by the Trypan blue dye exclusion assay.

### 4.9. Immunofluorescence Staining

Freshly collected goat PBMCs were treated with rFgTPx for 1 h at 37 °C in a humidified atmosphere with 5% CO_2_. The cells were pelleted by centrifugation at 300× *g* for 10 min and washed three times in PBS to remove unbound proteins. The goat PBMCs were fixed in 4% paraformaldehyde for 10 min at ambient temperature, washed three times in ice-cold PBS (5 min each), and then blocked with PBS containing 4% BSA for 1 h. The rFgTPx–treated or non–treated (control) goat PBMCs were incubated with primary rabbit anti–rFgTPx antibody (1:500) for 1 h at 37 °C. After incubation, the cells were washed three times in PBS and stained with Cy3-conjugated goat anti-rabbit IgG (1:500) (Beyotime, Haimen, Jiangsu, China). The cells were washed five times after being incubated with secondary antibody at 37 °C for 1 h. Hoechst 33,342 (Invitrogen, Eugene, OR, USA) was used to counterstain the cell nuclei. Immunofluorescence-labeled cell preparations were analyzed using a Zeiss laser scanning microscope (LSM710, Zeiss, Jena, Germany) with the 63× oil-immersion objective and Zeiss operating system associated with the ZEN imaging program.

### 4.10. Enzyme-Linked Immunosorbent Assays

The levels of cytokines (interleukin-2 (IL-2), IL-4, IL-10, and IL-17), transforming growth factor beta (TGF-β), and interferon gamma (IFN-γ) were measured using antibodies and standards provided in the goat enzyme-linked immunosorbent assay (ELISA) kits (Mlbio, Shanghai, China). Briefly, 100 µL of RPMI 1640 medium containing goat PBMCs (10^6^ cells/mL) was added into 96-well tissue culture plates (100 µL/well) and exposed to serial concentrations (5, 10, 20, and 40 μg/mL) of rFgTPx. PBMCs treated with medium only served as controls. After incubation at 37 °C and 5% CO_2_ for 72 h, the supernatant was collected, and cytokine levels were measured using ELISA kits in accordance with the manufacturer’s instructions.

### 4.11. Cell Proliferation Analysis

RPMI 1640 medium containing 10^6^ cells/mL were placed in a 96–well tissue culture plate (100 µL/well). The rFgTPx at 5, 10, 20, and 40 μg/mL concentrations was added to the respective wells, and wells containing medium only served as controls. The plates were placed in 5% CO_2_ incubator for 48 h. Then, 10 μL of CCK-8 reagent (Beyotime Biotechnology, Haimen, Jiangsu, China) was added. After incubation in dark for 4 h, the absorbance was measured at 450 nm (OD_450_) using a microplate reader (Bio-Rad Laboratories, Hercules, CA, USA). Cell proliferation index was calculated as follows: OD_450_ treated cells/OD_450_ control. 

### 4.12. Cell Migration Assay

Cell migration was investigated using a Millicell insert with 8 μm pores (Merck Millipore, Darmstadt, Hessen, Germany). Approximately 10^6^ cells/well were added into a 24-well tissue culture plate and stimulated with rFgTPx at 5, 10, 20, and 40 μg/mL concentrations in RPMI 1640 medium in a total volume of 1 mL/well. After incubation at 37 °C and 5% CO_2_ for 24 h, cells were harvested, and the cell number was adjusted to 1.5 × 10^6^ cells/mL. Then, 3 × 10^5^ cells (200 μL) were added into the upper chamber, while the bottom chamber of the inserts was loaded with 1300 μL of RPMI 1640 medium. After incubation for 2 h, the inserts were removed, and a Neubauer counting chamber was used to count the cells, which migrated through the membrane to the bottom chamber. The non-treated cells were used as a control. 

### 4.13. Measurement of Total Nitric Oxide (NO) Production

To measure the production of total NO in PBMC’s supernatant, a Griess assay (Abcam, Cambridge, MA, USA) was used as previously described [21]. The rFgTPx at the same concentrations stated above was used to stimulate goat PBMCs (10^6^ cells/mL) in DMEM medium. After 24 h, the culture supernatant was collected and processed using a Griess assay kit. A microplate reader (Bio-Rad Laboratories, Hercules, CA, USA) was used to measure absorbance values at 540 nm (OD_540_), and then the results were expressed as micromoles per liter. The non-treated cells served as control. 

### 4.14. Determination of Phagocytic Activity

The effect of rFgTPx on the phagocytic activity of PBMCs was investigated by measuring the uptake of FITC–dextran by monocytes, as previously described [21]. Briefly, 10^6^ monocytes were inoculated to a 24-well tissue culture plate and stimulated with rFgTPx (5, 10, 20, and 40 μg/mL) in RPMI 1640 medium for 48 h. Then the cells were collected and re-suspended in 100 μL of cold PBS, then 100 μL of 1 mg/mL FITC–dextran (Sigma, St Louis, MO, USA) were added. Cold PBS containing 2% FBS was used to stop the reaction. FACS Calibur analysis (BD Biosciences, San Jose, CA, USA) and FlowJo 7.6 software (Tree Star, Ashland, OR, USA) were used to collect and analyze the data. Cell phagocytosis index was determined by median fluorescence intensity (MFI) values. The blank control was set as 100%. The non–treated cells were used as the control. 

### 4.15. Cell Apoptosis Assay

PBMCs (10^6^ cells/mL) treated with rFgTPx (5, 10, 20, and 40 μg/mL) were incubated at 37 °C in a humidified atmosphere with 5% CO_2_. After 24 h, an annexin V–FITC kit (Miltenyi Biotec, Bergisch Gladbach, Nordrhein-Westfalen, Germany) was used to detect cell apoptosis. The stained cells were analyzed using flow cytometry (BD Biosciences, San Jose, CA, USA). Cells treated with PBS were used as control.

### 4.16. Statistical Analysis

Data analysis was performed using GraphPad Prism ver. 7 (GraphPad Software, San Diego, CA, USA). One-way ANOVA with a Dunnett’s test was performed to analyze the difference between groups. The difference was regarded as statistically significant when the *p*-value was <0.05. 

## 5. Conclusions

We provided the first molecular and functional characterization of FgTPx. Western blot showed that rFgTPx is recognized by the sera of infected goats and specific rabbit serum. When incubated with goat PBMCs, all concentrations of rFgTPx increased the secretion of IL-2, IL-4, IL-10, IL-17, TGF-β, and IFN-γ, but inhibited cell proliferation and migration, and reduced monocyte phagocytosis. Also, 20–40 μg/mL of rFgTPx increased the release of total NO, and 10–40 μg/mL of rFgTPx promoted cell apoptosis. Our data indicate that FgTPxs contribute to the immune evasion strategy employed by *F. gigantica* by modulating the functions of PBMCs, which represent the first line of defense against parasite infection. By modulating and inflicting damage on the effector cells of the immune system, FgTPx may modify the host capacity to counter *F. gigantica* invasion. Although the precise immunological mechanism remains to be elucidated, rFgTPx clearly impairs several functions of goat PBMCs and the subsequent development of adaptive immunity. Given the pervasive modulatory effects that FgTPx exhibited, understanding the exact functions of this effector protein may ultimately lead to the development of novel immunomodulatory interventions for the treatment of *F. gigantica* infection. Future studies should investigate the effect of a combination of ESPs (rFgTPx, rFg14-3-3e, and rFgRab10) on the induction of an immune response by goat PBMCs in vitro and to protective immunity in vivo.
